# Attrition Rates in HIV Viral Load Monitoring and Factors Associated With Overdue Testing Among Children Within South Africa’s Antiretroviral Treatment Program: Retrospective Descriptive Analysis

**DOI:** 10.2196/40796

**Published:** 2024-05-14

**Authors:** Ahmad Haeri Mazanderani, Lebohang Radebe, Gayle G Sherman

**Affiliations:** 1 Centre for HIV & STIs National Institute for Communicable Diseases National Health Laboratory Service Johannesburg South Africa; 2 Department of Paediatrics & Child Health Faculty of Health Sciences University of the Witwatersrand Johannesburg South Africa; 3 Paediatric HIV Diagnostics Division Wits Health Consortium, University of the Witwatersrand Johannesburg South Africa

**Keywords:** HIV, monitoring, viral load, suppression, overdue, retention, VL test, attrition, child, youth, pediatric, paediatric, sexually transmitted, sexual transmission, virological failure, South Africa, infant, adolescent, big data, descriptive analysis, laboratory data

## Abstract

**Background:**

Numerous studies in South Africa have reported low HIV viral load (VL) suppression and high attrition rates within the pediatric HIV treatment program.

**Objective:**

Using routine laboratory data, we evaluated HIV VL monitoring, including mobility and overdue VL (OVL) testing, within 5 priority districts in South Africa.

**Methods:**

We performed a retrospective descriptive analysis of National Health Laboratory Service (NHLS) data for children and adolescents aged 1-15 years having undergone HIV VL testing between May 1, 2019, and April 30, 2020, from 152 facilities within the City of Johannesburg, City of Tshwane, eThekwini, uMgungundlovu, and Zululand. HIV VL test–level data were deduplicated to patient-level data using the NHLS CDW (Corporate Data Warehouse) probabilistic record-linking algorithm and then further manually deduplicated. An OVL was defined as no subsequent VL determined within 18 months of the last test. Variables associated with the last VL test, including age, sex, VL findings, district type, and facility type, are described. A multivariate logistic regression analysis was performed to identify variables associated with an OVL test.

**Results:**

Among 21,338 children and adolescents aged 1-15 years who had an HIV VL test, 72.70% (n=15,512) had a follow-up VL test within 18 months. Furthermore, 13.33% (n=2194) of them were followed up at a different facility, of whom 3.79% (n=624) were in a different district and 1.71% (n=281) were in a different province. Among patients with a VL of ≥1000 RNA copies/mL of plasma, the median time to subsequent testing was 6 (IQR 4-10) months. The younger the age of the patient, the greater the proportion with an OVL, ranging from a peak of 52% among 1-year-olds to a trough of 21% among 14-year-olds. On multivariate analysis, 2 consecutive HIV VL findings of ≥1000 RNA copies/mL of plasma were associated with an increased adjusted odds ratio (AOR) of having an OVL (AOR 2.07, 95% CI 1.71-2.51). Conversely, patients examined at a hospital (AOR 0.86, 95% CI 0.77-0.96), those with ≥2 previous tests (AOR 0.78, 95% CI 0.70-0.86), those examined in a rural district (AOR 0.63, 95% CI 0.54-0.73), and older age groups of 5-9 years (AOR 0.56, 95% CI 0.47-0.65) and 10-14 years (AOR 0.51, 95% CI 0.44-0.59) compared to 1-4 years were associated with a significantly decreased odds of having an OVL test.

**Conclusions:**

Considerable attrition occurs within South Africa’s pediatric HIV treatment program, with over one-fourth of children having an OVL test 18 months subsequent to their previous test. In particular, younger children and those with virological failure were found to be at increased risk of having an OVL test. Improved HIV VL monitoring is essential for improving outcomes within South Africa’s pediatric antiretroviral treatment program.

## Introduction

South Africa has an estimated 8 million people living with HIV [1]. Although the national Prevention of Mother to Child Transmission program, launched in earnest in 2004, has reduced the vertical transmission rate to approximately 3%, there remain around 280,000 children and adolescents nationwide aged <15 years living with HIV. Furthermore, pediatric and adolescent populations living with HIV have far worse clinical outcomes than older age groups [2]. Modeling studies from South Africa estimate that while the proportion of the total population living with HIV who are diagnosed, those accessing antiretroviral therapy (ART), and those with viral suppression (<1000 RNA copies/mL of plasma [hereinafter “copies/mL”]) is 94%, 69%, and 63%, respectively, for pediatric populations, these proportions are considerably lower at 82%, 57%, and 39%, respectively [1]. Partly in response to the disparity between pediatric and adult treatment outcomes, the UNAIDS (Joint United Nations Programme on HIV/AIDS) launched the Global Alliance to End AIDS in Children by 2030 program, with South Africa officially having launched its chapter in 2023 [3].

Poor pediatric linkage to care, high attrition rates, and high mortality rates have been described from a number of cohort studies across the country [4-6]. Such studies support the identification of variables associated with attrition—this is considered an important step toward improving care. Virological nonsuppression has been identified as one such key risk factor for subsequent program loss from both urban and rural cohorts [7]. However, as variables associated with attrition may vary among regions and are likely to be influenced by a myriad of socioeconomic and political factors, additional mechanisms are urgently needed to not only identify children at risk of disengaging with care but also support the challenging process of re-engagement. Effective use of routine laboratory data offers opportunities toward this end.

The National Health Laboratory Service (NHLS) is the only clinical laboratory service provider within South Africa’s public health sector. As such, the NHLS Data Warehouses can potentially be leveraged for cost-effective disease surveillance and near–real-time reporting, with the advantage of not being restricted to facility-based monitoring as is the case with the national electronic patient monitoring system, TIER.Net. The ability of laboratory data to support the ready disaggregation of population-level data to provide a more nuanced understanding of program outcomes has been demonstrated for the pediatric HIV program, with younger children found to have markedly lower viral suppression rates than older children, and male patients being less likely to be suppressed than female patients across all age groups [8]. Furthermore, reporting of patient-identifying consolidated exceptions, both at district and facility levels, as indicated in the Results for Action reports of the National Institute for Communicable Diseases (NICD), a division of the NHLS, enables the timely identification of children in need of urgent follow-up based on HIV diagnostic and monitoring findings [9]. However, the potential for routine laboratory data for evaluating pediatric attrition in near real time, thereby supporting retention in care, has not previously been described.

In the following analysis, we aimed to evaluate HIV viral load (VL) monitoring within the pediatric HIV program by reporting on patient mobility and overdue VL (OVL) testing within 5 high-burden health districts in South Africa.

## Methods

### Study Design

This study is a retrospective descriptive analysis of routine laboratory data from South Africa’s public health sector, spanning a 4-year period. Data for children and adolescents aged <15 years living with HIV, who had undergone HIV VL testing between May 1, 2019, and April 30, 2020, from 152 facilities within 5 districts, were extracted from the NICD Data Warehouse. Test-level data were deduplicated to patient-level data by 2 sequential methods. The first one involved assigning a unique patient identifier (UPI) to all results by using a probabilistic record-linking algorithm, referred to in the text as the NHLS CDW (Corporate Date Warehouse) unique identifier (hereinafter referred to as the “CDW algorithm”), which links HIV tests performed nationally within the NHLS. This algorithm has been validated within the adult HIV program, with a reported sensitivity of 84% and incorrectly matching 0.3% of results [10]. Second, records were manually deduplicated by 4 data experts, according to a set of agreed upon rules listed in Multimedia Appendix 1 and referred to in the text as the manual deduplication UPI. Manual deduplication linked HIV tests for each patient within the 152 facilities only. Prior and subsequent tests were linked using both the CDW algorithm and manual deduplication identifiers. Prior tests from October 1, 2017, were linked to the cohort to describe the number of previous tests and number of previous high HIV VL (≥1000 copies/mL) results. Subsequent tests for an 18-month period up to October 31, 2021, were linked to the cohort to describe follow-up testing. An OVL test was defined as no subsequent VL test within 18 months of the last VL test, irrespective of the last HIV VL result. An 18-month period was informed by national ART guidelines, which recommend that all patients undergo HIV VL monitoring at least once every 12 months. While other similar analyses have used a 24-month period [11,12], an 18-month period (ie, considering a 6-month grace period) was deemed sufficient and supported the inclusion of data up until April 2020 at the time of analysis. Due to known limitations with linking infant tests using demographic data [13], analysis of OVL tests was restricted to children and adolescents aged 1-15 years.

### Setting and Laboratory Testing Methods

HIV viral load routine laboratory data were analyzed from 5 high-HIV-burden districts in South Africa considered by the National Department of Health as having the largest pediatric treatment gaps in the country, as determined from modeling data: City of Johannesburg and City of Tshwane in Gauteng Province, and eThekwini, uMgungundlovu, and Zululand in KwaZulu-Natal Province [14]. Data were restricted to patients who had undergone HIV VL testing at one of 152 designated facilities that have additional pediatric HIV support from District Support Partner organizations, representing 30% of all facilities and approximately two-thirds of all HIV VL testing performed for children and adolescents aged <15 years within these districts.

HIV VL monitoring is routinely recommended 6 months after ART initiation, with repeat testing performed at 12 months and 12-monthly thereafter if viral suppression is detected. The cutoff that defines suppression was revised in the 2019 national guidelines from <1000 copies/mL to <50 copies/mL, with patients with an unsuppressed VL requiring repeat testing at 3 months after a thorough assessment and management of the cause of an elevated VL [15].

Within the NHLS, HIV VL testing is performed using plasma samples at centralized laboratories using either the Cobas HIV-1 Test (Roche Diagnostics) or the Abbott RealTime HIV-1 assay (Abbott Molecular, Inc).

### Statistical Analysis

Variables associated with the last HIV VL finding per patient, tested between May 1, 2019, and April 30, 2020, are reported; these include age group (<1, 1-4, 5-9, or 10-14 years), sex (male, female, or unknown), VL test result (<50, 50-999, or ≥1000 copies/mL), district type (metropolitan districts classified as urban versus nonmetropolitan districts classified as rural), and facility type (hospital versus primary health care facility). The number of patients according to each deduplication step is described by comparing the number and percentage decrease of patients reported through manual deduplication of UPIs and deduplication performed using the CDW algorithm per variable. The proportion of people with an OVL was evaluated for both deduplication methodologies. Median (IQR) time to the subsequent HIV VL test is reported.

A multivariate logistic regression analysis was performed to identify whether specific variables were associated with an OVL. The data for the model were restricted to children aged ≥1 year with a known gender. In addition, patients were excluded if they only had a single test, with no linked prior or subsequent test. Variables were included in the model if their associated univariate Pearson chi-square *P* value was <.15. Backwards feature selection was used to select relevant variables, and the Hosmer-Lemeshow goodness-of-fit test was used to evaluate the final model. Statistical analysis was performed using R (version 3.6.3; The R Foundation) on a 64-bit Windows PC.

### Ethical Considerations

The National Institute for Communicable Diseases has ethics approval for communicable disease surveillance and analysis of routine laboratory data by the Human Research Ethics Committee of the University of the Witwatersrand (M160667; M210752), with the requirement for informed consent having been waived. All analysis with patient-identifiable data was performed on a secure password-protected server located on the NHLS campus.

## Results

### Data Deduplication

**Figure 1:**
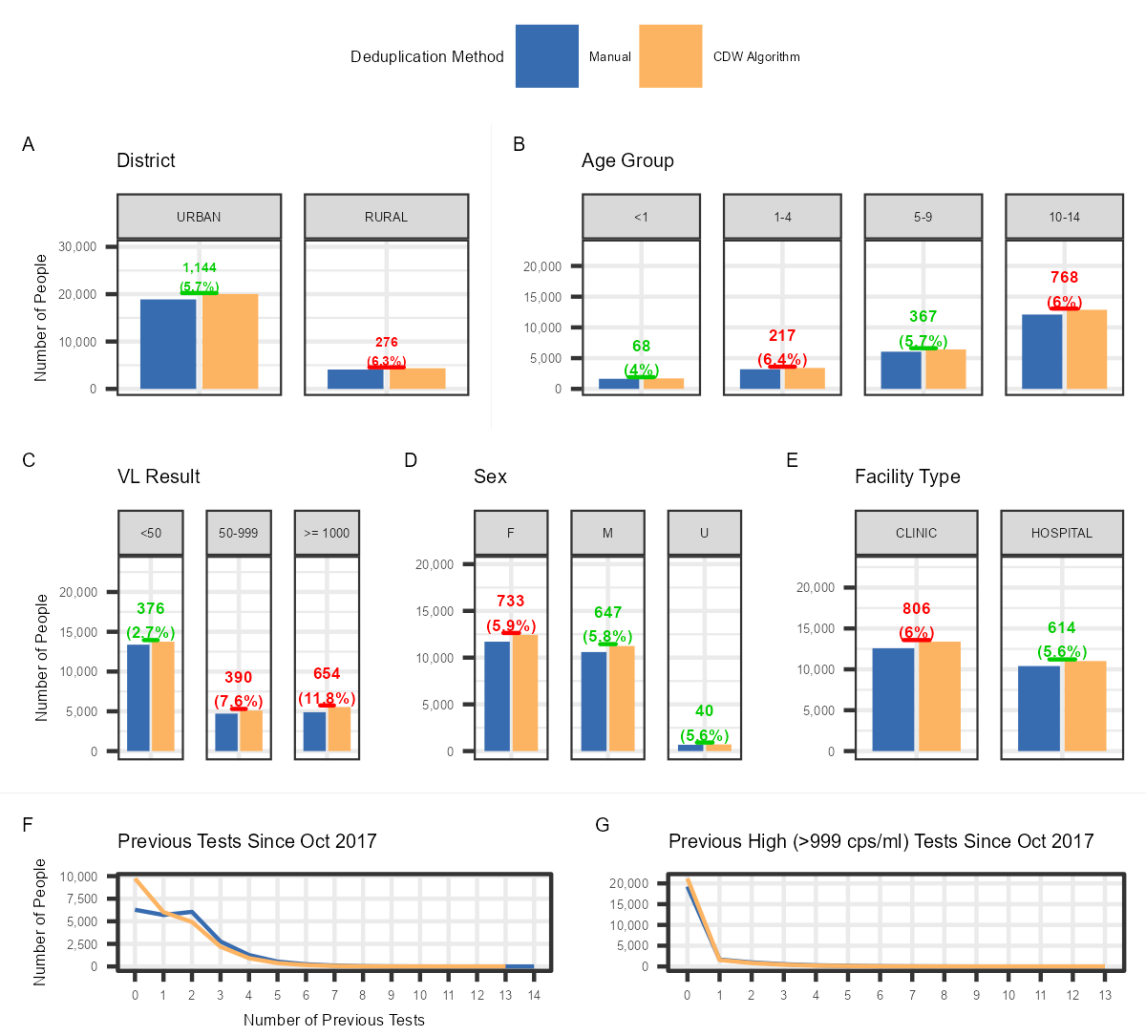
Number and percentage deduplication of test-level to patient-level data using 2 unique patient identifiers (UPIs) among children and adolescents aged <15 years with an HIV viral load (VL) test between May 2019 and April 2020 from the City of Johannesburg, City of Tshwane, eThekwini, uMgungundlovu, and Zululand by (A) district, (B) age group, (C) VL test result, (D) sex, (E) facility type, (F) number of previous linked tests, and (G) number of previous linked high VL (>999 RNA copies/mL of blood) findings. CDW: Corporate Data Warehouse; cps/ml: RNA copies/mL of blood.

A total of 29,822 HIV VL tests, registered among children and adolescents aged <15 years, were carried out between May 2019 and April 2020 at 152 facilities. According to the CDW algorithm, these corresponded to 24,398 patients, while the manually deduplicated UPIs corresponded to 22,978 patients, representing a deficit of 5.82% (n=1420). This deficit was associated with considerable variation among variables except for sex and facility type (Figure 1). Facilities in the urban districts had a greater deficit in the number of participants (n=1144) than those in rural districts (n=276); however, this represented a smaller deficit in the proportion of people (5.7% vs 6.3%, respectively; Figure 1A). In terms of age, the 1-4–year age group had the highest percentage of deduplication at 6.4% (n=217), while the <1-year age group had the lowest proportion of deduplication at 4.0% (n=68; Figure 1B). There was considerable variation in deduplication among HIV VL result categories: those with a VL of <50 copies/mL decreasing by 2.7% (n=376), those with a VL of 50-999 copies/mL decreasing by 7.6% (n=390), and those with a VL of ≥1000 copies/mL decreasing by 11.8% (n=654; Figure 1C). In terms of sex and facility type, the variation was minimal, with a 5.9% (n=733) reduction among females and a 5.8% (n=647) reduction among males (Figure 1D), and a 6.0% (n=806) reduction for clinics compared with a 5.6% (n=614) reduction for hospitals (Figure 1E). In terms of the number of linked previous tests, there was a marked decreased in number of patients with a single HIV VL test and those with no prior high (≥1000 copies/mL) HIV VL finding (Figures 1F and 1G).

Differences were also observed in the number and proportion of patients with an OVL test after manual deduplication when compared with the CDW algorithm. According to the CDW algorithm, 22,690 children and adolescents aged 1-15 years had undergone an HIV VL test between May 2019 and April 2020 within the 152 designated facilities, of whom 9685 (42.68%) had an OVL test. After manual deduplication, 21,338 children and adolescents were identified, of whom 5826 (27.3%) had an OVL test, representing a reduction of 39.85% (n=3859) of patients considered to have an OVL test.

### Follow-Up HIV VL Testing and Movement Across Facilities

Among the manually deduplicated data set of 21,338 children and adolescents aged 1-15 years who had undergone an HIV VL test between May 2019 and April 2020, a total of 54,160 tests were registered at 771 different facilities. Considering the results of the first test registered with each patient, 11,889 (55.7%) had a VL of <50 copies/mL, 4416 (20.7%) had a VL of 50-999 copies/mL, and 5033 (23.6%) had a VL of ≥1000 copies/mL. In terms of location, 17,341 (81.3%) tests were first registered in an urban district and 3997 (18.7%) were registered in a rural district. At the time of the first test, there were 3303 (15.5%) patients aged 1-4 years, 6126 (28.7%) patients aged 5-9 years, and 11,909 (55.8%) patients aged 10-14 years.

**Figure 2:**
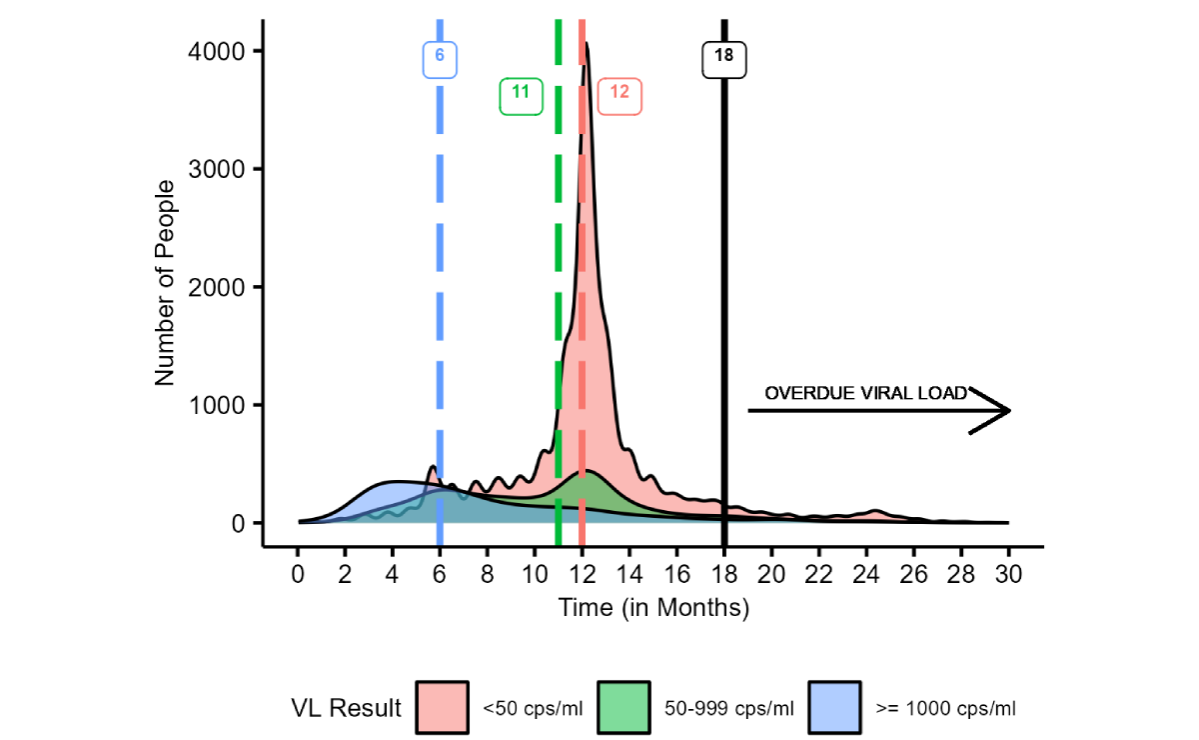
Time to follow-up HIV viral load (VL) testing (in months) by VL findings, with medians reported per result category based on manual deduplication of unique patient identifiers among children and adolescents aged 1-15 years having undergone an HIV VL test between May 2019 and April 2020 from the City of Johannesburg, City of Tshwane, eThekwini, uMgungundlovu, and Zululand. cps/ml: RNA copies/mL of blood.

Of the 21,338 children and adolescents, 77.16% (n=16,465) of them had undergone a follow-up VL test, of whom 72.70% (n=15,512) had undergone a follow-up HIV VL test within 18 months and 4.47% (n=953) had a follow-up test after 18 months. Overall, 14,363 (87.2%) patients were followed up at the same facility, 1963 (11.9%) were followed up at 2 facilities, 120 (0.7%) were followed up at 3 facilities, 17 (0.1%) were followed up at 4 facilities, and 2 (0.01%) were followed up at 5 facilities. In total, 2102 (12.8%) patients were followed up at a different facility, of whom 604 (3.7%) were in a different district and 270 (1.6%) were in a different province. The median time to subsequent testing varied by VL result (Figure 2). For those with a VL of <50 copies/mL (n=9944), the median time of subsequent testing was 12.1 (IQR 11.1-13.1) months. For those with a VL finding of 50-999 copies/mL (n=3505), the median time to subsequent testing was 11.2 (IQR 7.2-12.8) months, and for those with a VL finding of ≥1000 copies/mL (n=3016), the median time to subsequent testing was 6.5 (IQR 4.2-10.3) months. For those with a single VL finding of ≥1000 copies/mL (n=1056), the median follow-up time was 7.1 (IQR 4.7-11.4) months, whereas for those with a repeat VL of ≥1000 copies/mL (n=1960), the median follow-up time was 6.1 (IQR 4.0-9.6) months. The change in the national guidelines in 2019, whereby the threshold for viral suppression was revised from <1000 to copies/mL to <50 copies/mL, was associated with a biphasic pattern of time to follow-up among patients with a finding between 50 and 999 copies/mL.

**Figure 3:**
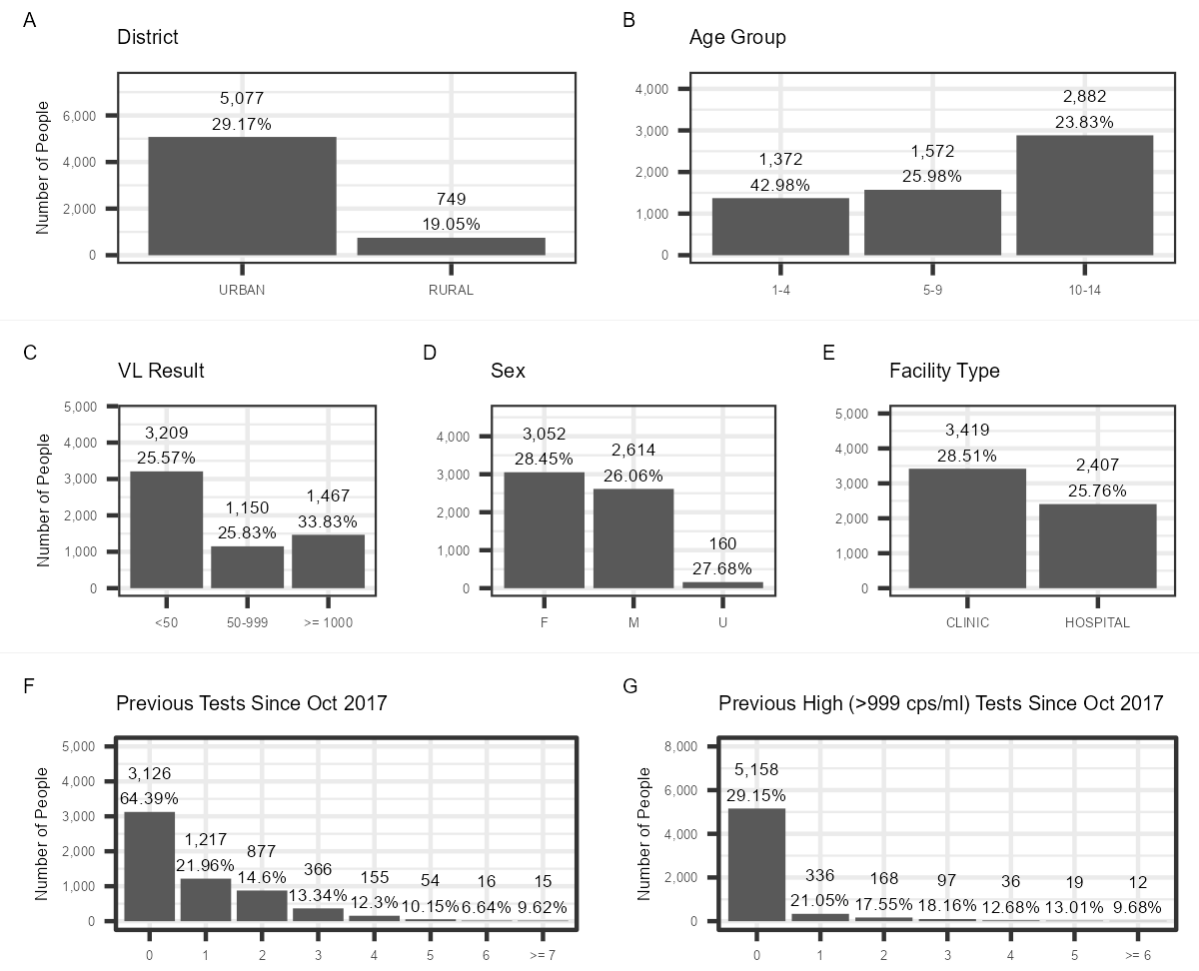
Number and percentage deduplication of test-level to patient-level data after manual deduplication for overdue viral load (VL) tests among children and adolescents aged 1-15 years having undergone an HIV viral load test between May 2019 and April 2020 from the City of Johannesburg, City of Tshwane, eThekwini, uMgungundlovu, and Zululand by (A) district, (B) age, (C) VL findings, (D) sex, (E) facility type, (F) number of previous linked tests, and (G) number of previous linked high HIV VL (>999 RNA copies/mL of blood) findings. cps/ml: RNA copies/mL of blood.

Urban districts had a greater number and proportion of people (n=5077, 29.17%) with an OVL test than rural districts (n=749, 19.05%; Figure 3A). In terms of age, the 1-4–year age group had the highest proportion of OVL tests at 42.98% (n=1372), while the 10-14–year age group had the lowest at 23.83% (Figure 3B). However, given the number of patients, this age category also had the highest number of people with an OVL test (n=2882). In terms of HIV VL test results, 25.57% (n=3209) of patients with a VL of <50 copies/mL and 25.83% (n=1150) of those with a VL of 50-999 copies/mL had an OVL test, while those with a VL of ≥1000 copies/mL accounted for the greatest proportion of OVL tests, at 33.83% (n=1467; Figure 3C). In terms of sex, a slightly higher proportion of females (n=3052, 28.45%) had an OVL test than males (n=2614, 26.06%; Figure 3D). Furthermore, a slightly higher proportion of people who underwent VL testing at a clinic had an OVL (n=3419, 28.51%) than those who underwent testing at a hospital (n=2407, 25.76%; Figure 3E).

In terms of the number of linked previous tests, the largest proportion of people with an OVL test was observed among patients with no linked prior tests (n=3126, 64.39%; Figure 3F). Not only did having at least one prior linked VL test considerably reduce the proportion of people with an OVL test, but also this proportion generally decreased as the number of previous linked tests increased. This ranged from a peak of 21.96% (n=1217) for those with one prior VL test to a trough of 6.64% (n=16) for those with 6 prior tests. This pattern was replicated in the number of previous high VL findings (>999 copies/mL; Figure 3G).

### OVL Tests by Age and Outcome

**Figure 4:**
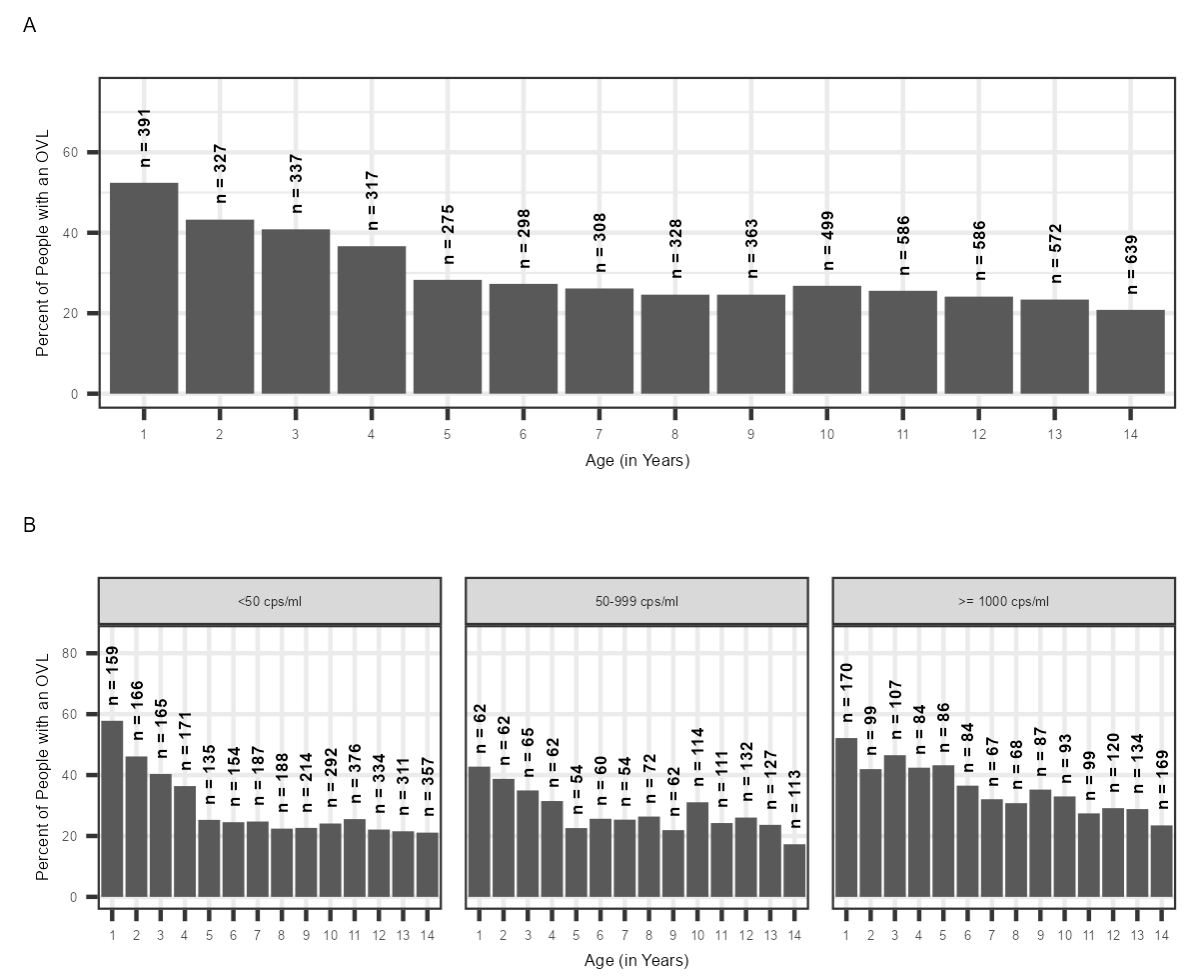
Number and percentage of people per age group (in years) with an overdue HIV viral load (VL) test among children and adolescents aged 1-15 years having undergone an HIV VL test between May 2019 and April 2020 from the City of Johannesburg, City of Tshwane, eThekwini, uMgungundlovu, and Zululand (A) overall and B) by HIV VL findings. OVL: overdue viral load.

As the age of the patient increased, the proportion of patients having an OVL test decreased, ranging from a peak of 52% among 1-year-olds to a trough of 21% among 14-year-olds (Figure 4A). This trend was observed across HIV VL result categories, with a higher proportion of OVL tests among patients with a VL of ≥1000 copies/mL (Figure 4B). Because of differences in population size among the age groups, the absolute number of patients with an OVL test varied, with high numbers of patients in the older age range having an OVL test (Figure 4A).

### Variables Associated With an OVL Test

Multivariate logistic regression analysis was performed to determine variables associated with an OVL test. Only patients with 2 or more HIV VL tests were included in the model to control for limitations associated with deduplication of the test-level data (details of patients excluded from the model are provided in Multimedia Appendix 1). In total, 16,648 patients were included in the model, of whom 1554 (9.33%) had an OVL test and 15,094 (90.67%) had a follow-up VL test within 18 months of their last VL test. The 7 categorical variables included in the model, owing to their associated univariate Pearson chi-square test *P* value of <.15, were age, current VL finding, any single high VL (≥1000 copies/mL excluding current results), 2 consecutively determined high VLs, number of previous tests, district type, and facility type (Table 1).

**Figure 5:**
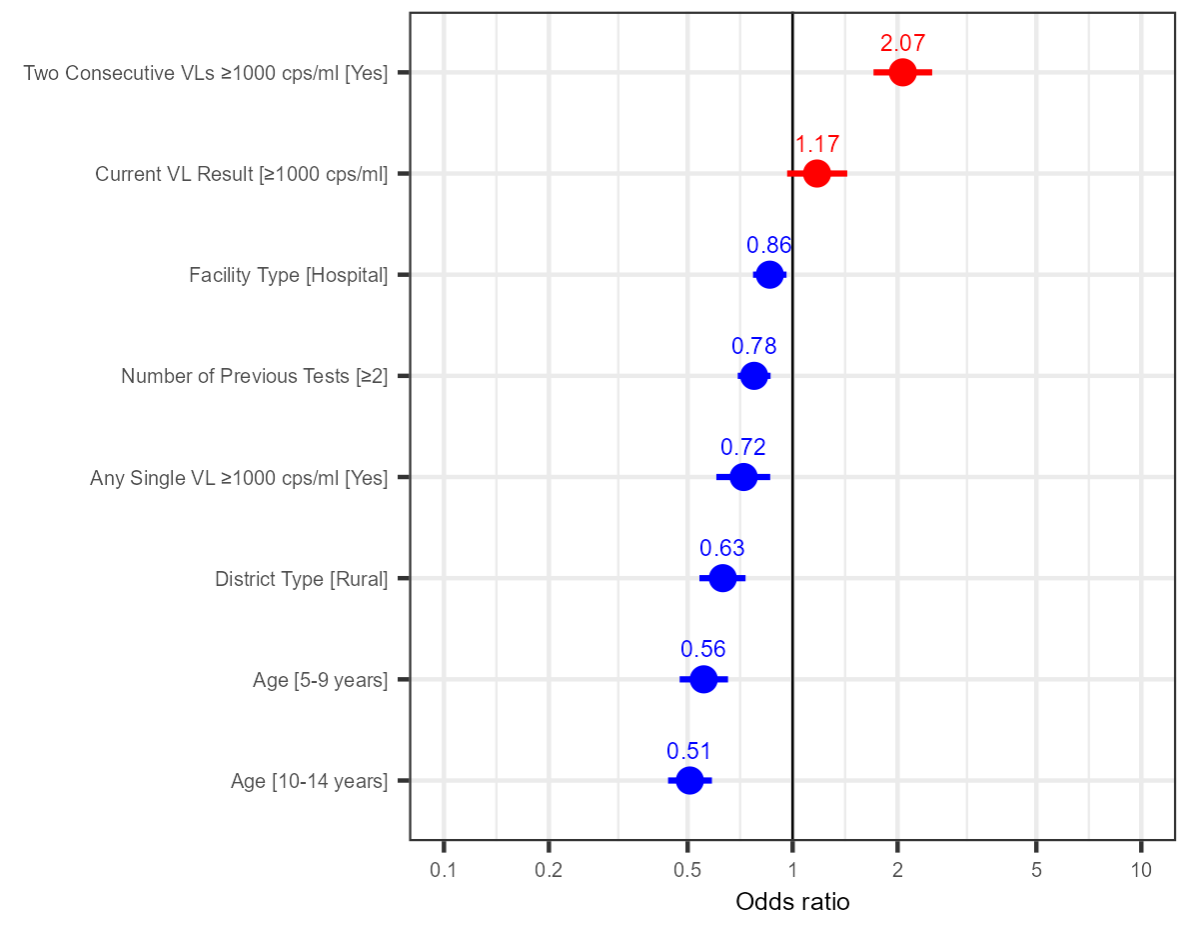
Multivariate logistic regression odds ratios to evaluate variables associated with overdue HIV viral load (VL) testing among children and adolescents aged 1-15 years having undergone an HIV VL test between May 2019 and April 2020 from the City of Johannesburg, City of Tshwane, eThekwini, uMgungundlovu, and Zululand. cps/ml: RNA copies/mL of blood.

The Hosmer-Lemeshow goodness-of-fit *P* value for the model was .90. On multivariate analysis, 2 consecutive (prior and current) HIV VL test results of ≥1000 copies/mL were associated with a significantly increased odds of having an OVL test. Conversely, the odds of having an OVL test were significantly low among patients examined at a hospital (rather than a clinic), those with ≥2 previous tests (compared to those with <2 tests), those with a history of a prior high HIV VL finding (excluding the current findings), those examined in a rural district, and those with an older age. A current VL finding of ≥1000 copies/mL was associated with increased odds but was not significant in the final model (Figure 5 and Table 2).

**Table 1:** Variables used in multivariate logistic regression analysis to evaluate overdue HIV viral load testing among children and adolescents aged 1-15 years having undergone an HIV viral load test between May 2019 and April 2020 from the City of Johannesburg, City of Tshwane, eThekwini, uMgungundlovu, and Zululand.

Variables		Overdue HIV viral load test, n (%)			Overall, n (%)
		No (n=15,094)	Yes (n=1554)		
**Age (years)**					
	1-4	1744 (84.74)	314 (15.26)	2058 (12.36)	
	5-9	4343 (91.03)	428 (8.97)	4771 (28.66)	
	10-14	9007 (91.73)	812 (8.27)	9819 (58.98)	
**Facility type**					
	Clinic	8238 (90.02)	923 (9.98)	9251 (55.57)	
	Hospital	6766 (91.47)	631 (8.53)	7397 (44.43)	
**Facility district**					
	Urban	12,035 (89.95)	1345 (10.05)	13,380 (80.37)	
	Rural	3059 (93.60)	209 (6.40)	3268 (19.63)	
**Current HIV viral load test outcome (RNA copies/mL of plasma)**					
	<1000	12,308 (91.08)	1205 (8.92)	13,513 (81.17)	
	≥1000	2786 (88.87)	349 (11.13)	3135 (18.83)	
**Any high HIV viral load test result**					
	False	10,436 (90.90)	1045 (9.10)	11,481 (68.96)	
	True	4658 (90.15)	509 (9.85)	5167 (31.04)	
**Number of previous HIV viral load tests**					
	<2	5886 (89.03)	725 (10.97)	6611 (39.71)	
	≥2	9208 (91.74)	829 (8.26)	10,037 (60.29)	
**Consecutive high HIV viral load test results**					
	False	13,773 (91.27)	1318 (8.73)	15,091 (90.65)	
	True	1321 (84.84)	236 (15.16)	1557 (9.35)	

**Table 2:** Results of the univariate Pearson chi-square Test and multivariate logistic regression analysis of variables associated with an overdue HIV viral load test among children and adolescents aged 1-15 years who underwent an HIV viral load test between May 2019 and April 2020 from the City of Johannesburg, City of Tshwane, eThekwini, uMgungundlovu, and Zululand.

Variables		*P* value (univariate Pearson chi-square test)	Outcomes of multivariate logistic regression analysis		
			Adjusted odds ratio	95% CI	*P* value
**Age (years; reference: 1-4 years)**		<.001	—^a^	—	—
	5-9		0.56	0.47-0.65	<.001
	10-14		0.51	0.44-0.59	<.001
**Facility type (reference: clinic)**					
	Testing at a hospital	.002	0.86	0.77-0.96	.008
**Facility district (reference: urban)**					
	Rural	<.001	0.63	0.54-0.73	<.001
**Current HIV viral load test result (RNA copies/mL of plasma; reference: <1000 RNA copies/mL of plasma)**					
	≥1000	<.001	1.17	0.96-1.43	.11
**Any high HIV viral load test result (reference: false)**					
	True	.13	0.72	0.60-0.86	<.001
**Number of previous HIV viral load tests (reference: <2)**					
	≥2	<.001	0.78	0.70-0.86	<.001
**Consecutive high HIV viral loads (≥1000 RNA copies/mL of plasma; reference: false)**					
	True	<.001	2.07	1.71-2.51	<.001

^a^Not applicable.

## Discussion

### Principal Results

In this analysis, which included over 20,000 children and adolescents living with HIV who are aged 1-15 years from 5 districts in South Africa, we found that over one-fourth of individuals had no repeat HIV VL test within 18 months from their previous test, suggesting considerable attrition within the pediatric population. Furthermore, among patients who underwent follow-up testing, delays in repeat testing were found among those with an elevated VL of >1000 copies/mL, similar to delays described for adult cohorts in South Africa [16]. As viraemia is associated with both patient morbidity and the emergence of drug-resistant HIV strains, these findings highlight the need to improve VL monitoring and quality of care among children and adolescents within the ART program.

Individual variables associated with an OVL test included patients of a younger age, having more recently initiated ART (as determined by <2 prior HIV VL tests), having a repeatedly high HIV VL of >1000 copies/mL, being tested in an urban district rather than a rural district, and being tested at a clinic rather than a hospital. Factors accounting for higher rates of OVL in urban districts and clinics require further investigation but may be related to shifting patient populations (due to migration to or use of multiple clinics in urban areas) and access to multidisciplinary teams, respectively. Importantly, a consecutive high HIV VL finding of >1000 copies/mL was found to be a key risk factor for having an OVL test, whereas a single high VL result was protective, suggesting that patients initially identified with viraemia are successfully returned to care but if they remain unsuppressed (ie, have virological failure) they are at a high risk of subsequently disengaging with care.

Among those children and adolescent patients who had undergone additional VL testing, a fair amount of mobility was observed. In total, 13.33% of patients were followed up at a different facility, 3.79% were followed up in a different district, and 1.71% were followed up in a different province. As the time to follow-up for this analysis was restricted to only 18-30 months per patient, it is likely a far higher proportion of mobility can be expected over the course of a child’s treatment history. This highlights limitations with South Africa’s TIER.Net HIV treatment database, which uses facility-level monitoring and hence necessarily underestimates retention of HIV care [17-19]. Furthermore, the extent to which children who have initiated ART in South Africa cross national borders and resume HIV care outside of their countries has yet to be described, as well as the number of deaths among this cohort (on account of restricted access to the national death register).

One of the notable findings of this analysis was the impact that additional manual deduplication of an already automated deduplicated data set had on programmatic outcomes. As South Africa has yet to effectively implement a national UPI, automated record linking algorithms are applied to laboratory data in an attempt to deduplicate test-level data sets to represent patient-level data. However, our findings suggest that without additional manual deduplication, analysis of public health laboratory data using the NHLS CDW algorithm would have overestimated the proportion of patients aged 1-15 years with an OVL by at least 40% (even though the data set was reduced by only an additional 6% after manual deduplication). This highlights the need to incorporate a national health UPI within the health system, including the laboratory information system and NHLS Data Warehouses, to effectively monitor the ART program.

### Limitations

A number of important limitations need to be considered regarding these findings. The NHLS CDW data set was manually deduplicated at the facility level (for the 152 facilities evaluated in this analysis) without the opportunity to compare findings against a gold-standard data set. Although we report that 13% of patients with a repeat HIV VL test were followed up at a different facility, this is likely an underestimation on account of the reduced ability to accurately merge the test results of patients who were examined at multiple facilities. This may have been a contributing factor to the reason why the urban regions were associated with a higher rate of OVL testing, if patients examined in the urban areas are more likely to be followed up at multiple facilities within or even outside of the district. Additionally, patients may be retained in care but not have repeat HIV VL tests in accordance with the guidelines, for instance, on account of challenges with accessing pediatric phlebotomy services [20]; hence, the OVL testing rate may overestimate program attrition. This is likely to have been further exacerbated during the course of the COVID-19 pandemic, which impacted maternal and child access to HIV services [21]. As the multivariate analysis evaluating variables associated with OVL testing was restricted to patients with ≥2 VL tests (to ensure goodness-of-fit of the model by increasing the likelihood that if additional testing was performed, it would be linked via the deduplication methodology), this may have also influenced the findings by not including patients who were only followed-up for a single HIV VL test and hence disengaged with care soon after ART initiation. Lastly, as our analysis was restricted to 30% of facilities within 5 of the 52 national districts, the results may not be nationally representative, especially considering that the districts included in this study are known to have a high pediatric HIV disease burden.

### Conclusions

In summary, linking population clinical data with laboratory data in a legal manner, which protects individuals’ rights, has the potential to improve patient outcomes for multiple health programs. A functional UPI for health care would enable improved longitudinal surveillance of children and adolescents living with HIV by using routine laboratory data for accurate, timely identification of patients who need to be returned to care, including infants. In the interim, 1-4–year-olds and those with a sustained VL of ≥1000 copies/mL should be managed as especially high risk for loss to follow-up.

## Appendix

Multimedia Appendix 1 Manual deduplication rules.

## Abbreviations

ART: antiretroviral therapy
CDW: Corporate Data Warehouse
NHLS: National Health Laboratory Service
NICD: National Institute for Communicable Diseases
OVL: overdue viral load
UNAIDS: Joint United Nations Programme on HIV/AIDS
UPI: unique patient identifier
VL: viral load

## Data Availability

The data sets analyzed in this study are not publicly available because they contain information that could compromise participant privacy, including patient first and last names, gender, locations, and tests conducted. Please contact the South African National Health Laboratory Service [22] for data requests.
