# Supplementary material for: Attrition Rates in HIV Viral Load Monitoring and Factors Associated With Overdue Testing Among Children Within South Africa’s Antiretroviral Treatment Program: Retrospective Descriptive Analysis
Source: JMIR Public Health Surveill. 2024 May 14;10:e40796. doi: 10.2196/40796 (PMC11134236; doi:10.2196/40796)
Supplement: Multimedia Appendix 1 [file publichealth_v10i1e40796_app1.docx]

**Multimedia Appendix 1. Manual Deduplication Rules**

Patient lists were extracted for each of the 152 UB-II facilities. Test-level data were de-duplicated using the NHLS CDW record-linking algorithm to represent patient-level data, with the last HIV-related test result presented (i.e. HIV VL, PCR, ELISA or CD4 count). These patient lists were then manually de-duplicated if three of four key identifiers (first name, surname, date of birth, folder number) were considered the same. The rules for considering variables to be the same are provided below. Records which did not meet criteria for matching criteria but were considered similar were shared with the District Support Partners at facilities. The partner organizations were requested to confirm if these records belonged to the same patient by evaluating patient files to determine whether HIV test results could be linked.

**Rules for considering variable to be the same**

| **Variable** | **Rule for Matching Variable** |
| --- | --- |
| First Name | Up to three letter/numeral differences provided same pattern |
| Surname | Up to three letter/numeral differences provided same pattern |
| Date of Birth | Allow for difference in either day, month, or year; or transposition of day, month, and/or year |
| Folder Number | Up to one letter and/or numeral difference |

**Supplementary Table 1. Number and Percent of People Excluded from Multivariate Logistic Regression Due to No Prior or Subsequent Linked Tests**

| **Age (in Years)** | **Number of People with No Linked Tests** | **Total Number of People (Per Age)** | **Percent of Total (%)** |
| --- | --- | --- | --- |
| 1 | 295 | 721 | 40.92 |
| 2 | 247 | 727 | 33.98 |
| 3 | 256 | 798 | 32.08 |
| 4 | 227 | 837 | 27.12 |
| 5 | 183 | 936 | 19.55 |
| 6 | 202 | 1 061 | 19.04 |
| 7 | 220 | 1 144 | 19.23 |
| 8 | 229 | 1 292 | 17.72 |
| 9 | 255 | 1 427 | 17.87 |
| 10 | 373 | 1 820 | 20.49 |
| 11 | 404 | 2 231 | 18.11 |
| 12 | 408 | 2 380 | 17.14 |
| 13 | 398 | 2 389 | 16.66 |
| 14 | 415 | 2 997 | 13.85 |
